# Temporal differences of onset between primary skin lesions and regional lymph node lesions for tularemia in Japan: a clinicopathologic and immunohistochemical study of 19 skin cases and 54 lymph node cases

**DOI:** 10.1007/s00428-012-1246-7

**Published:** 2012-05-17

**Authors:** Shigeyuki Asano, Kikuo Mori, Kazuki Yamazaki, Tetsutaro Sata, Takayuki Kanno, Yuko Sato, Masaru Kojima, Hiromi Fujita, Yasushi Akaike, Haruki Wakasa

**Affiliations:** 1 Department of Pathology, Iwaki Kyoritsu General Hospital, 16 Kusehara, Mimaya-machi, Uchigo, Iwaki, 973-8555 Japan; 2 Department of Pathology, National Institute of Infectious Diseases, Tokyo, Japan; 3 Department of Pathology, Dokkyo University School of Medicine, Tochigi, Japan; 4 Ohara Laboratory Institute, Fukushima, Japan; 5 Department of Pathology, Shimotsuga General Hospital, Tochigi, Japan; 6 Tohoku Bunka Gakuen University, Sendai, Japan

**Keywords:** Tularemia, Primary skin lesions, Regional lymph node lesions, Temporal differences of onset

## Abstract

For tularemia, a zoonosis caused by the gram-negative coccobacillus *Francisella tularensis*, research of the relation between skin lesions and lymph node lesions has not been reported in the literature. This report describes skin lesions and lymph node lesions and their mutual relation over time for tularemia in Japan. Around the second day after infection (DAI), a subcutaneous abscess was observed (abscess form). Hand and finger skin ulcers formed during the second to the fourth week. Subcutaneous and dermal granulomas were observed with adjacent monocytoid B lymphocytes (MBLs) (abscess–granulomatous form). From the sixth week, large granulomas with central homogeneous lesions emerged diffusely (granulomatous form). On 2–14 DAI, *F. tularensis* antigen in skin lesions was detected in abscesses. During 7–12 DAI, abscesses with adjacent MBLs appeared without epithelioid granuloma (abscess form) in regional lymph nodes. During the second to fifth week, granulomas appeared with necrosis (abscess–granulomatous form). After the sixth week, large granulomas with a central homogeneous lesion (granulomatous form) appeared. *F. tularensis* antigen in lymph node lesions was observed in the abscess on 7–92 DAI. Apparently, *F. tularensis* penetrates the finger skin immediately after contact with infected hares. Subsequently, the primary lesion gradually transfers from skin to regional lymph nodes. The regional lymph node lesions induced by skin lesion are designated as dermatopathic lymphadenopathy. This study revealed temporal differences of onset among the skin and lymph node lesions.

## Introduction

Tularemia is a zoonosis caused by the gram-negative coccobacillus *Francisella tularensis*, the etiologic agent of the disease [1–5]. Soken Honma, a physician in Mito, Ibaraki Prefecture, Japan, described tularemia as “hare meat poisoning” (1837) [3] in the oldest report of the disease. In California, USA, McCoy (1911) [6] reported a plague-like disease affecting squirrels. Ohara and Kitamura [3, 7] confirmed its transmissibility to humans. *F. tularensis* was named after Francis and the town in which the bacterium was isolated: Tulare, California, USA (1921) [6]. In Japan, Hachiro Ohara established the Ohara Institute in Fukushima for the active study of tularemia (1925) [3]. Japanese armed forces (1932–1945) and the U.S. Army (1950–1960) undertook studies to develop tularemia bacteria for use as a biological weapon [1, 8, 9].

After bioterrorism with anthrax in 2001, the Centers for Disease Control (CDC) classified tularemia into the most dangerous pathogen group, category A, along with smallpox and anthrax [10]. Thereafter, cases of tularemia have been reported worldwide [2, 11, 12], but it has become an exceedingly rare disease in Japan. Although the incidence of this disease has decreased, its details must be reviewed because of its potential for use in acts of bioterrorism [1, 8, 10, 13] and because of the danger it presents as an infectious disease transmitted by animals [1, 3, 7].

Research of lymph node lesions [7] has been more common for this disease, but skin lesions and the relation between primary skin lesions and lymph node lesions has not described in the literature. This report of cases in Japan describes primary skin lesions and lymph node lesions and their mutual relation over time for tularemia in terms of clinicopathology and immunohistochemistry.

## Materials and methods

Between 1950 and 1965, data of 19 skin cases and 54 lymph node cases were collected at the Ohara Laboratory Institute, Fukushima Japan. All patients files reserved at the Institute were used for this research. These files contained clinical data including symptoms, intimate contact day from infected hares, onset day and biopsy day for each patient. In addition, laboratory examinations such as serum agglutinin and skin test against *F. tularensis* showed positive.

Sections cut from 10 % formalin-fixed paraffin-embedded skin and lymph node samples were stained with hematoxylin–eosin (HE), Giemsa, periodic acid-Schiff (PAS), and Watanabe's silver impregnation.

For immunohistochemical examination, formalin-fixed tissue sections (4-μm thick) were deparaffinized in xylene and redehydrated in graded alcohols and distilled water. All tissue sections were incubated in 0.01 M citrate buffer (pH 6.0) (Koso Chemical Co., Ltd., Tokyo, Japan) using standard microwave or autoclave heating technique for 15, 20 min, respectively. Furthermore, immunohistochemical examination of deparaffinized sections was performed using an automated stainer (Ventana Medical Systems Inc., Arizona, USA) according to the manufacturer's instructions. Then, they were mounted with Malinol mounting (Muto Pure Chemicals Co. Ltd., Tokyo, Japan).

The panel of antibodies against CD 3 (Roche Diagnostics Corp., Ventana Medical Systems Inc.), CD 4, CD10 (Nichirei Corp., Tokyo Japan), CD 5, CD 8, CD 20, CD 30, CD 68 (all Dako, Carpinteria, Ca, USA), CD 83 (Novocastra Laboratories Ltd., U.K.), CD 163 (Lab Vision Corp., USA), CD 204 (Dr. Takeya, Kumamoto, Japan), Langerin (Novocastra Laboratories Ltd.), S-100 (Dako), D2-40 (Nichirei Corp.), Fascin, HLA-DR, IgG, IgA, IgM, κ, λ (all Dako), and anti-*F. tularensis* antibody (Dr. Hotta, NIID, Japan) were used. Sections with known reactivity to the assayed antibodies served as positive controls. Negative controls consisted of each case tissue incubated with normal mouse serum instead of the antibody against *F. tularensis* (Table 1).

**Table 1:** Antibodies used in this immunohistochemical study

Antibody	Clone	Specificity	Source	Clonality	Retrieval	Dilution
CD3	2GV6	T cells	Roche	M	Mic	1:1
CD4	1 F6	Helper/inducer T cells	Nichirei	M	Mic	1:1
CD5	CD5/5456	T cells	Dako	M	Mic	1:100
CD8	C8/144B	Cytotoxic/suppressor T cells	Dako	M	Mic	1:1
CD10	56 C6	CALLA, Immature B cells, germinal center B cells	Nichirei	M	Mic	1:1
CD20	L26	B cells	Dako	M	Mic	1:1
CD30	Ber-H2	Activated B cells	Dako	M	Mic	1:100
CD68	KP1	Macrophage, plasmacytoid T cells	Dako	M	Mic	1:50
CD83	1H4b	Dendritic cells, Langerhans cells	Novo	M	Mic	1:20
CD163	10D6	Macrophage scavenger receptor	Lab	M	Mic	1:50
CD204		Macrophage scavenger receptor	^a^	M	Mic	1:1
Langerin	12D6	Langerhans cells	Novo	M	Mic	1:100
S-100		Langerhans cells, melanocyte, Schwan cells	Dako	P	Non	1:1
D2-40	D2-40	Lymph vessel	Nichirei	M	Mic	1:1
Fascin	55 K-2	Dendritic cells, interdigitating reticulum cells	Dako	M	Mic	1:50
HLA-DR	TAL.1B5	Langerhans cells, Macrophages, B cells, activated T cells	Dako	M	Mic	1:25
IgG		IgG	Dako	P	Non	1:1
IgA		IgA	Dako	P	Mic	1:1
IgM		IgM	Dako	P	Mic	1:1
κ	R10-21-f3	κ	Dako	M	Non	1:1
λ	N10/2	λ	Dako	M	Non	1:1
*F.tularensis*		*F.tularensis*	^b^	P	Auto	1:1

*Roche* Roche Diagnostics, Arizona, USA, *Nichirei* Nichirei, Tokyo, Japan, *Dako* Dako, Ca, USA, *Novo* Novocastra, UK, *Lab* Lab Vision, USA

*M* monoclonal, *P* polyclonal, *Mic* microwave, *Auto* autoclave, *Non* non treated

^a^Anti-CD204 antibody and ^b^anti-*F*. *tularensis* antibody were supplied from Dr. Takeya and Dr. Hotta, respectively

## Results

### Clinical findings

Clinical features of the skin lesions (19 cases) and lymph node lesions (54 cases) are presented in Tables 2 and 3 and almost all patients had visited the doctor for common cold-like symptoms such as a sudden high fever (38–40°C) with chill, headache, back pain, cough, and sore throat. Most patients were male agricultural workers. Almost all patients had been infected during skinning of hares and during cooking of infected hare meat. Skin lesions and lymphadenopathy mainly included finger skin and subcutaneous lesions and regional axillary and elbow lymph nodes, respectively. There were no fatal cases.

**Table 2:** Summary of clinical findings of skin lesions, 19 cases

Age distribution	19–69	Mean 43 years, median 44 years
Male:female ratio	4:1	
Occupation	Farmer	13 cases (68 %)
	Charcoal maker	2
	Other	4
Day after infection	1–96 days	Mean 30 days, median 19 days
Site	Skin (hand finger)	4 cases
	Subcutaneous (axillary, elbow)	14
	Unknown	1

**Table 3:** Summary of clinical findings of lymphadenopathy, 54 cases

Age distribution	8–69		Mean 39 years, median 37 years
Male:female ratio	4:1		
Occupation	Farmer		40 cases (74 %)
	Charcoal maker		3
	Carpenter		2
	Other (teacher, woodcutter, charcoal maker, hotel worker) furrier, child)		9
Day after infection	6–133 days		Mean 33 days, median 24 days
Site	Axillary (76 %)	Left	21
		Right	21
		Bilateral	4
	Elbow (7 %)	Left	1
		Right	3
	Mandible		1
	Unknown		3

### Pathological findings

#### Skin lesion

**Fig. 1:**
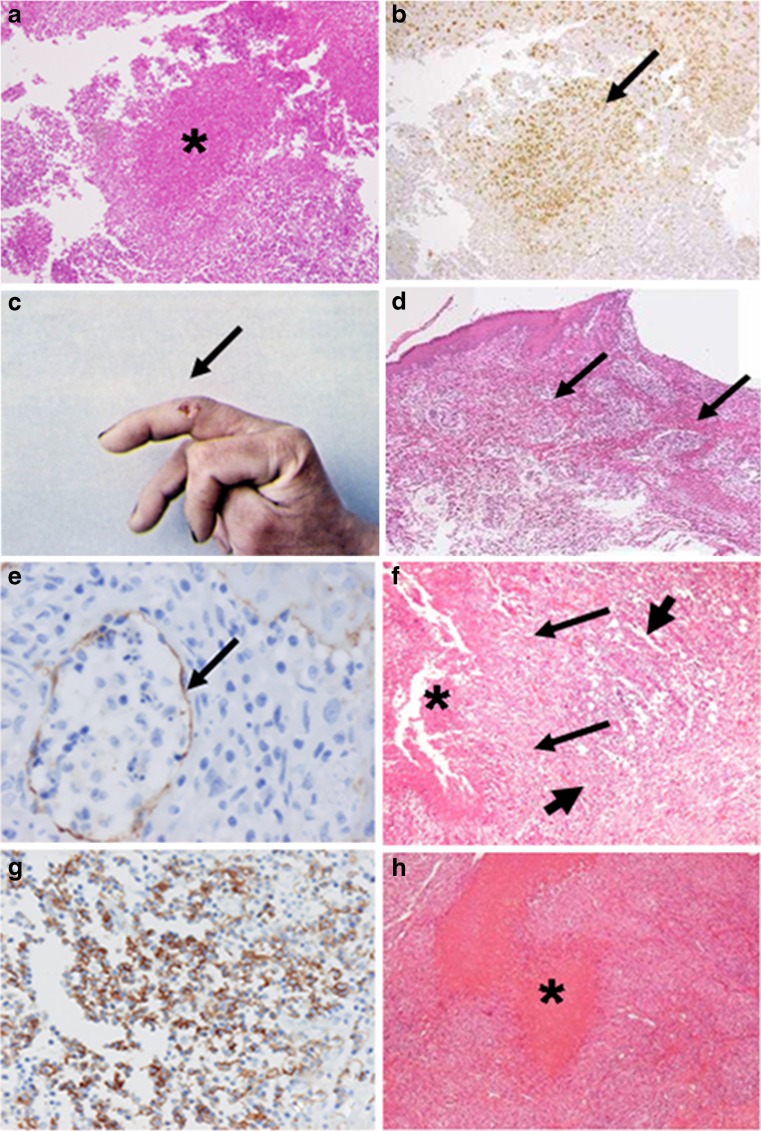
Skin and subcutaneous. **a** Central necrosis and abscess (*asterisk*) of subcutaneous area with marginal lymphocytes (4 DAI). **b** Immunostaining. *F. tularensis* antigens (*arrow*) were detected mainly in abscesses and necrotic areas (4 DAI) (anti-*F. tularensis* antibody). **c** Skin ulcer (*arrow*) of the right middle finger (14 DAI). **d** Various sizes of granulomas (*arrow*) at ulcer base of dermis. **e** Lymphocytes, apoptotic cells, and macrophages were observed within marked distended lymph vessels (*arrow*) (30 DAI). (Anti-D2-40 antibody). **f** Epithelioid granuloma (*arrow*) in dermis with central necrosis (*asterisk*) was adjacent to CD 20+ lymphocyte aggregation (*bold arrow = italic*). **g** Enlargement of **f**. Many CD 20+ lymphocytes (monocytoid B lymphocytes; MBLs) aggregated near the granuloma (19 DAI). **h** Large irregularly shaped epithelioid granuloma with central homogeneous lesion (*asterisk*) in dermis. No antigen was detected in the lesion (75 DAI)

In the early phase (1–8 days after infection, DAI), no ulceration was found. Many inflammation-related cells such as lymphocytes and plasma cells were observed, along with cell debris and necrosis without neutrophils in deeper dermis and subcutaneous regions under the superficial dermis (abscess form) (Fig. 1a). *F. tularensis* antigens were detected mainly in abscess and necrotic areas (Fig. 1b). Many S-100+, CD68+, CD83+, Fascin+, and CD163+ cells were found in dilated lymph vessels of dermal papillae. Furthermore, irregularly shaped abscesses and many S-100+ and Langerin+ dendritic cells were apparent in dilated lymph vessels around abscess and necrotic subcutaneous sites. Mainly CD20+ B cells are scattered near the abscess.

In the later phase, 14–20 DAI, ulcers appear (Figs. 1c, d). Marked dilated lymph vessels are apparent at the bottom of ulcers and dermal papillae where lymphocytes, dendritic cells, and apoptotic cells were phagocytized by macrophages (Fig. 1e). Subcutaneous areas show several immature granulomas with radiative patterns of epithelioid cells (abscess–granulomatous form) (Fig. 1f). Giant cells and inflammation were observed. Many S-100+, CD68+, CD163+, CD204+, Fascin+, and T cells were found in and around granulomas.

Aggregations of monocytoid B lymphocytes (MBLs) were adjacent to the granulomas (Fig. 1g).

Dilated lymph vessels become more numerous during 28–42 DAI and similar cells to those in the previous phase appear at the bottom of the ulcer of dermis. Numerous granulomas are also observed not only in subcutaneous areas but also in superficial dermis. In addition, numerous multinucleated giant cells appear near the granulomas.

In the last phase, skin ulcers are scarred after around the sixth week. During 51–96 DAI, many irregularly shaped fused epithelioid granulomas with central homogeneous lesions are observed in subcutaneous and dermis areas (granulomatous form) (Fig. 1h). Similar cells to those in the previous phase appear in and around granulomas. Giant cells are observed occasionally at epithelioid granulomas. Aggregations of MBLs, adjacent to the granulomas, were also observed in this phase. Many S-100+, CD68+, and CD163+ cells were also observed in marked distended lymph vessels of dermal papillae.

**Fig. 2:**
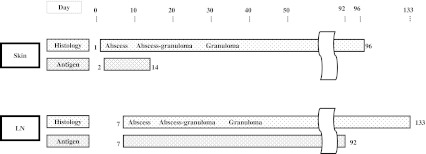
Histology and *F. tularensis* antigen of skin and lymph node. *F. tularensis* antigen was detected during 2–14 DAI, and 7–92 DAI, in abscess and necrotic area of skin and lymph node, respectively. *Day*, days after infection (numbers of columns denote days); *LN*, lymph node; *Antigen*, antigen for *F. tularensis*; Histology was classified into three forms: abscess, abscess–granulomatous, and granulomatous [7]

The *F. tularensis* antigen of the skin lesion was detected only in the abscess, except with granuloma at 2–14 DAI (Fig. 2).

#### Lymph node lesion

**Fig. 3:**
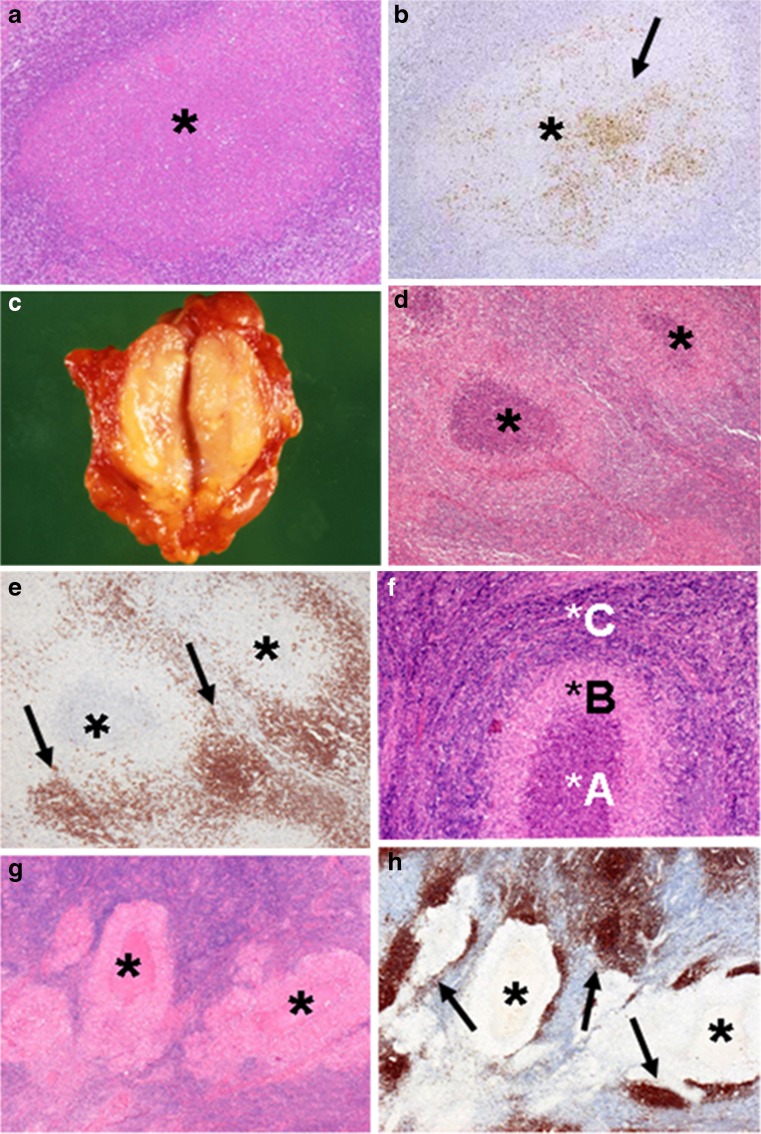
Abscess form lymph node lesion (10 DAI). **a** Abscess and necrosis lesion (*asterisk*) without epithelioid granuloma located in the paracortex of a lymph node (10 DAI). **b** *F. tularensis* antigens (*arrow*) were mainly present in abscesses and necrosis lesions (*asterisk*) (10 DAI). (Anti-*F. tularensis antibody).* **c** Abscess–granulomatous form lymph node lesion (14 DAI). Marked swelled axillary lymph node on the cut section. **d** Central abscess and necrosis lesion (*asterisk*) was surrounded by thick epithelioid cell granuloma (14 DAI). **e** CD20+ monocytoid B lymphocytes (MBLs) aggregations (*arrow*) were adjacent to granuloma. *Asterisk*, abscess and necrosis lesion (14 DAI). **f** CD4/CD8 ratio was 2.3 (* *A*), 1.1 (* *B*), and 2.0 (* *C*) in abscess–granulomatous form of lymph node. **g** Granulomatous form lymph node lesion (92 DAI). Large irregular granuloma with central homogeneous lesion (*asterisk*). Very few antigens were detected in abscesses. **h** CD20+ cells (MBLs) aggregations (*arrow*) were adjacent to the outer layer of epithelioid cell granuloma. *Asterisk*, central homogeneous lesion

During 7–12 DAI, sinus histiocytosis and follicular hyperplasia appeared. Small abscesses and mononuclear cells appeared (abscess form) (Fig. 3a). *F. tularensis* antigens were detected mainly in abscess and necrotic areas (Fig. 3b). Adjacent to abscesses, MBLs aggregation was observed. Some cases show marked periadenitis because of acute inflammation.

During the second to fifth week after infection, small epithelioid granulomas with central necrosis were apparent beneath the capsular area and medulla of lymph nodes (abscess–granulomatous form) (Figs. 3c, d). The epithelioid cells of the granulomas occasionally showed a radiative pattern. Many S-100+, CD68+, CD163+, CD204+, Fascin+, HLA-DR+, and T cells were found in and around granulomas.

The MBLs were adjacent to the granuloma, as in an earlier phase (Fig. 3e). Regarding lymphocyte distribution in and around granulomas, CD4/CD8 T cell ratios were 2.3, 1.1, and 2.0, respectively, at abscesses, granulomas, and outer layers of granulomas (Fig. 3f). Furthermore, numerous multinucleated giant cells are scattered in granulomatous lesions.

After the sixth week, many large granulomas with central homogeneous lesions resembling tuberculous nodes (granulomatous form) were present. They were mutually fused, forming irregularly shaped granulomas (Fig. 3g). Similar cells to those in the previous phase appear in and around granulomas. Many MBLs were observed as adjacent to granulomas, as noted for the prior phase (Fig. 3h). In addition, many multinucleated giant cells were observed in granulomatous lesions.

*F. tularensis* antigen was observed from 7–92 DAI in the abscess, except that with granuloma (Fig. 2).

## Discussion

From their pathological study of the tularemia, Kitamura et al. [7] described lymph node and skin phenomena. According to their report, lymph node lesions are classifiable with time into three forms: abscess form, abscess–granulomatous form, and granulomatous form. Skin ulcers form in the first week and scar tissue formation is observed in the second to fifth week after onset. Healing of skin lesions might be more rapid than that of lymph nodes [7].

Our study revealed that the primary skin lesions, especially in subcutaneous lesions, induced by *F. tularensis* occurred from 2 DAI and that lymph node lesions developed during 7–14 DAI. The time difference of onset was apparent among these skin and lymph node cases.

Molecular biological analysis showed that DNA sequences of *F. tularensis* from the infected hare coincided with that of regional lymph node of the patient [in prepare]. It may be concluded that *F. tularensis* penetrates intact human skin after direct contact with an infected hare. Subsequently, it is transported via the skin lymphatic stream to subcutaneous regions and then to regional lymph nodes [14, 15]. The most common skin ulcers occurred on the finger skin up to the second week after infection, and scars formed up to the sixth week in this study. Many antigen presenting cells (APCs) such as histiocytes, leukocytes, lymphocytes, and Langerhans cells were observed within dilated lymph vessels in the primary skin lesion. Many recruited APCs at the subcutaneous region or lymph node, contributing to production of abscesses and granuloma formation. During this period, granulomas with marked inflammation were formed at the base of the ulcer, and *F. tularensis* was detected only at abscesses. As described above, regional lymph node lesions induced by skin disease are designated as dermatopathic lymphadenopathy (DPL) [16]. The primary skin lesion gradually transfers from the skin to regional lymph nodes [14, 17].

Dermatopathic lymphadenopathy (DPL) is a commonly paracortical hyperplasia of a regional lymph node induced by chronic skin diseases such as erythroderma and mycosis fungoides. It persists even after distinct skin lesions have disappeared by the time of lymph node biopsy [16]. In general, the pathological finding of DPL is characterized by the presence of eosinophils, histiocytes, lymphoblasts, and Langerhans cells appearing as a pale area in the paracortex in the lymph node. In contrast, in tularemic lymph nodes, cells of such kinds do not appear because the primary skin lesions are acute dermatitis induced by *F. tularensis* [7]. Based on the discussion presented above, tularemic lymphadenopathy can be regarded as DPL of an acute type.

Secondary skin lesions on the hand, leg, and neck caused by an allergic reaction to bacteria are papular or papulovesicular eruptions that occur around 10 DAI. They might occur bilaterally, symmetrically, or as widely distributed, and can take 2 weeks to subside. Erythema nodosum and erythema multiforme are also secondary skin lesions. *F. tularensis* was also detected from erythema multiforme vesicle fluid [18, 19]. However, secondary skin lesions do not produce ulcers as primary skin lesions do [20, 21].

Earlier reports have described that *F. tularensis* antigen was detected only in the abscesses of skin and lymph node lesions [14]. The long-lasting antigen against *F. tularensis* in the lymph nodes might contribute to the sustained presence of serum antibodies. Furthermore, CD4+ T cells, which affect B cells to stimulate antibody production, were present in greater numbers in granuloma than CD8+ T cells were. The factors described above contributed to antibody production. However, the antigen was apt to diminish after mature granuloma formation.

In the early phase of tularemia, numerous neutrophils, monocytoid B cells (MBLs), histiocytic cells, T cells, S-100+, Langerin+, CD83+, CD163+ dendritic cells are found in lesions. An MBL cluster was occasionally observed adjacent to a microabscess from the abscess forming phase. During abscess and granuloma formation, macrophages might recruit MBLs with subsequent infiltration of neutrophils, followed by necrosis and eventual granuloma formation [22]. Furthermore, inflammatory cells such MBLs, CD68+, CD163+, CD204+ macrophages, S-100+, Langerin+, CD83+, CD163+ dendritic cells and T lymphocytes can be regarded as playing an important role for granuloma formation in tularemia, as in cat scratch disease [23].

It is occasionally difficult to distinguish tularemia from tuberculosis because they have similar granulomas. Tularemia, which has clinically acute inflammation, begins with much more rapid progress than that of tuberculosis. Regarding histologic characteristics of tularemia, the epithelioid cell layer is generally thicker than that of tuberculosis, with disappearance of argyrophilic fibers in the center of the granulomas of tularemia. Moreover, the nuclei of giant cells in tularemia are fewer than in tuberculosis [7].

Recently, rapid assay for detection of *F. tularensis* in formalin-fixed paraffin-embedded lymph nodes has become available for differential diagnosis by polymerase chain reaction (PCR) [19, 24]. As a biosecurity issue, it is necessary to design a molecular diagnosis system for *F. tularensis* to distinguish strains of subsp. *holarctica*, a common strain in Japan [25], from strains of other subspecies including strains of the highly virulent subsp. *tularensis* [2, 26].
